# Care service supply’s impact on household consumption inequality in the context of “one old and one young” families

**DOI:** 10.3389/fpubh.2025.1419294

**Published:** 2025-04-01

**Authors:** Bolin Dong, Huan Liu

**Affiliations:** ^1^School of Marxism, Tongling University, Tongling, China; ^2^School of Social Science, Soochow University, Su Zhou, China

**Keywords:** one old and one young, consumption inequality, family, older adults, dual care

## Abstract

**Introduction:**

With aging and family nuclearization, providing care for older adults and children has become a global issue. This study empirically examines the impact of care service supply on household consumption inequality.

**Methods:**

Data from the China Family Panel Studies are analyzed using the ordinary least squares estimation method.

**Results:**

First, childcare and older adult care significantly impact household consumption inequality by 0.23 and 0.35%, respectively. Further, their impacts on household consumption inequalities at different inequality levels show gradually increasing trend. Moreover, significant group heterogeneity is observed in the impact coefficients. Second, under the dual care pressures for childcare and older adult care, the supply of dual-care services significantly affects household consumption inequality by 0.05%.

**Conclusion:**

Care service supply has an income effect; that is, the supply of family care services affects household consumption inequality by influencing household income inequality. Policy considerations include improving the precision of social welfare and assistance policies, and enhancing the supply mechanism of care services.

## 1 Introduction

The demand for care is increasing. The International Labour Organization notes that, as of 2015, the number of people in need of care worldwide was approximately 2.1 billion, including 1.9 billion under the age of 15 and 200 million older adults.^1^ With the increasing demand for care services albeit slow growth in supply, care services supply has become a global problem and even a global “care crisis” (1). With China’s aging population and the rapid development of disabilities, the demand for older adult care is increasing and care services supply has become an increasingly prominent issue (1). By the end of 2021, the population aged 65 and over in China reached 200.56 million, accounting for 14.20% of the total population, indicating that the country has become an aging society with the proportion of the population aged 65 and over being between 14 and 20%.^2^ According to the National Health Commission, China has 40 million older adults with disabilities among its 250 million older adult population.^3^ Moreover, the proportion of older adults suffering from disabilities in China with more than mild disability is increasing annually, with a total disability ratio of nearly 30% (2).

Extant research on the supply of care services focuses on the care for older adults. There are obvious national differences in the development of care services in the world. For example, in some developed countries in Europe and North America, the welfare of children is relatively developed, and the care service system for the older population is relatively sound, so the whole care service supply system is relatively perfect. However, for developing countries such as China, under the realistic background of rapid population aging and gradual decline of fertility rate, the care service system for older people is still in the stage of gradual development, while the child care service is in the stage of exploration, which shows the vulnerability of the whole care service supply system. However, with the implementation of China’s family planning policy, the emergence of the “one old and one young” family structure has also led to an increasing demand for childcare services. Most studies explore childcare issues from the perspective of intergenerational care, whereas relatively few examine the comprehensive impact of care services on families. Care service providers can be divided into formal (social care) and informal care (family care). This study mainly considers the informal care provided by core family members at the family level, or family care services.

Eliminating inequality has become one of the important indicators of China’s social and economic development. The report of the 19th Communist Party of China (CPC) National Congress and outline of the 14th Five-Year Plan both set the goal of “narrowing the income gap among residents.” The report of the 20th CPC National Congress further noted the lofty goal of promoting “common prosperity, “with reducing inequality among groups being an essential part of narrowing the income gap among residents and achieving common prosperity. Inequality has always been an important research topic in economics. It mainly manifests as inequality and heterogeneity in income, consumption, and wealth (3). Income increases ultimately require consumption to increase utility. As such, consumption is more closely linked to resident welfare. Indeed, scholars are increasingly concerned about the level of consumption and its heterogeneity to reflect the true differences in residents’ welfare levels (3, 4). Meanwhile, as an important part of the household labor supply, care service supply has not been fully compensated for by society (except for nursing allowance or childcare allowance policies, such as those in some pilot areas of long-term care insurance in China). Further, its actual supply can differ across households due to family structure differences, which can cause income inequality or consumption inequality. Specifically, consider a young-type family structure. These families only have demand for childcare services because the intergenerational care provided by the parent generation can alleviate childcare pressures on such families. However, old-type families have both parents and children who require care. Hence, the care pressure on the core labor force of such families is much higher than that of young-type families. “Care pressure” is defined by the comprehensive proportion of family care for children and the older adults according to the pressure impact of care service behavior on different families. If there is no family care, we believe that the family care pressure is the least; There is one kind of care for the older adults or children, and we think its family care is general; For families with two kinds of care services, we believe that the family pressure is the greatest.

Under China’s goal of common prosperity, effectively releasing the potential for household consumption and promoting different households to achieve common prosperity are important issues facing China’s social development. In particular, the supply of family care services under with the family structure’s transformation is an important link. First, the supply of family care services is an important component of social labor. While its social value should be recognized, solutions to enhance care services supply still need further investigation. Second, the supply of family care services creates group heterogeneities owing to differences in family structure. Then, measuring the resulting differences in family welfare is worthy of further exploration. Accordingly, this study explores the impact of family care service supply on household consumption inequality considering family structure transformation. Further, this article focuses on consumption inequality under the dual care pressures in “one old and one young” families to identify the practical dilemmas faced by such families. The resulting insights can be valuable for designing appropriate social security policies and improving care service policies.

This article makes two contributions: First, considering the “one old and one young” family care service supply, this article examines the impact of family care service supply on household consumption inequality. Thus, we enriched research on this relationship, and expand the research field of household consumption inequality. Second, focusing on the dual care of “one old and one young” families, we explore the impact and transmission mechanism of care service supply on household consumption inequality and consumption structure, providing empirical support for the impact of family care service supply on household consumption inequality, and expanding research on family care service supply.

The following chapters are arranged as follows: Literature review and theoretical analysis, methods and data, results, discussion and conclusions.

## 2 Literature review

Care service supply generally refers to the long-term care from family members or society to meet the care needs and improve the quality of life of individuals who lack full self-care abilities due to young age, illness, or old age. As such, intuitively, care service demand comprises daily care needs arising from these individuals’ lack of self-care abilities. The costs and time spent in the care process constitute the cost of care demand, or the burden of care services supply. Regarding older adult care service supply and demand, research reveals that with the acceleration of population aging, both developed and developing countries are seeing an increasing proportion of older adult care service supply costs to GDP (5–9). For example, Zhou and Alan found that the cost of family care for older adults with disabilities has increased significantly in the five major regions of China (10). However, improvements in the health status of older adults can only reduce their social care needs and not improve their demand for family care services (11–13). For childcare needs, social care is gradually replacing traditional family care (14, 15). Moreover, with population aging and declining birth rates, grandparental care has made up for the lack of social care but still cannot meet the demand for care services (16).

Further, an extensive literature has examined the impact of the care service supply. First, given its importance for older adults with disabilities, family care positively affects their physical and mental health, but negatively affects the physical and mental health of caregivers (17–19). Moreover, care service supply is an important part of family burden, which significantly affects family labor supply decision-making to some extent. With an aging population, this trend further weakens the ability of low-income families to increase their income. Research shows that in terms of older adult care, high care burdens not only affects the physical and mental health of caregivers, but also more strongly affects the health of female caregivers in families (20–22). Meanwhile, care service supply has a greater effect on reducing the quality of life of female caregivers (23). Second, regarding childcare services, research mainly focuses on the effects of intergenerational care and female childcare (24–26). Specifically, intergenerational care not only promotes family labor supply, but also benefits families with two children. However, due to differences in family compatibility, intergenerational care has different effects on family members who work in non-agricultural and agricultural fields. As women bear more responsibility for childcare, it inhibits their employment probability and working hours. Intergenerational care can effectively alleviate the pressure on women who give birth (27, 28).

**Figure 1:**
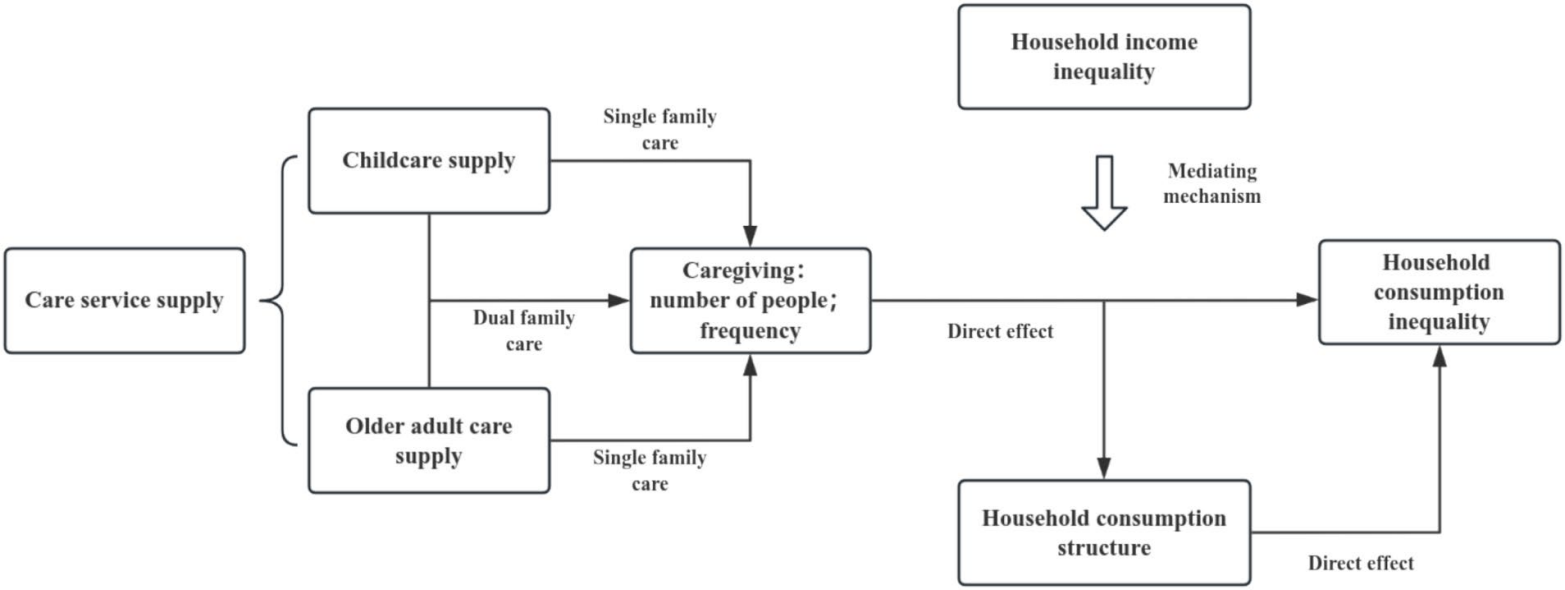
Theoretical frameworks.

Nonetheless, research on the perspective of “one old and one young” deserves further attention. The existing studies have conducted rich research on the care of older people and the minor children of the family, as well as its impact on the current situation of the family. However, the focus of existing studies is on the existence of care, and the investigation of care behavior, care frequency and family comprehensive care differences are not clearly involved, so this article analyzes the literature on care service supply from two aspects: the impact of care service supply on household consumption, and the underlying transmission mechanism. Simultaneously, the research hypotheses are proposed. The specific theoretical framework is shown in Figure 1:

## 3 Theoretical analysis

### 3.1 Impact of care service supply on household consumption

Care service supply can significantly increase total household consumption and promote the household consumption structure upgrading (29, 30). However, for families with high-intensity care, the care service supply suppresses consumption through the punishment and health effects. That is, care labor will reduce the supply of non care labor for family members, and weakening the overall income level of their families, thereby reducing household consumption levels (31). Extant research on the transmission mechanism of care service supply to household consumption mainly focuses on three aspects. First, care service supply affects the household consumption level by affecting the health level of family care members. For example, older adult care can effectively reduce the health risks for older adults, thereby reducing their associated medical expenditures and promoting non-medical consumption in households (32, 33). Second, family care service supply will reduce the labor supply of family members, which in turn affects their household income and consumption levels, resulting in an income penalty effect due to family care service supply (34–36). Third, family care service supply affects family consumption in the form of intergenerational transfer; specifically, rational reciprocity via family care service supply helps promote family consumption structure adjustments by core family members, and thus, improves the total consumption level (34, 37). For example, grandchild care is conducive to promoting the overall consumption level of middle-aged and older adult families, and proportion of leisure consumption expenditures, reducing the proportion of consumption expenditure toward basic needs, and affecting family consumption through children’s emotional support for parents, family social interaction level, and Internet use (38). Fourth, family care service supply has heterogenous effects on households with different consumption levels. Intergenerational care has similar effects. Specifically, compared to households with medium or high levels of consumption, intergenerational care has a more significant impact on the consumption expenditure of households with lower levels of consumption (34). An extensive literature has explored the impact of care service supply on the health of caregivers and women of childbearing age from both macro and micro perspectives, as well as its impact on household consumption and income inequality.

However, many areas worthy of investigation remain. For example, research on the supply of care services mainly focuses on the perspectives of older adult or intergenerational care, while relatively limited work on the supply of care services from the perspective of “one old and one young” families. There is relatively little discussion on the trends and inequalities in household consumption for these families. Research still focuses on household consumption or household income inequality. Understanding the impacts on household consumption inequality and the transmission mechanisms are also important.

In general, existing studies have examined the significant impact of family care service on household consumption and consumption structure from the perspective of the total amount of family care service, and gradually deepened to the impact of family care service on consumption levels and inequality of different family structures. Existing research conclusions show that family care service will enhance the degree of consumption inequality among different families. For example, due to the increase of family care service, the income and consumption level of families requiring care will be reduced to a certain extent, while the level of non caring families will remain unchanged or improve, resulting in the expansion of consumption inequality among families. At the same time, studies have shown that the difference in care service will lead to changes in the family consumption structure, especially increasing the proportion of family survival consumption expenditure, which will lead to inequality and changes in the family consumption structure. At the same time, family care work includes care services such as taking care of children and the older adults. Accordingly, the first and second hypotheses are proposed as follows:

*H1*: The provision of care services, including childcare and older adult care, negatively affects household consumption inequality.

*H2*: The supply of care services affects the household consumption structure, and has heterogenous effect on different types of consumption inequalities.

### 3.2 Transmission mechanism of care service supply on household consumption inequality

Studies have rarely focused on the impact of the family care service supply on household consumption inequality, and more often focused on the impact on household income inequality. For example, some studies have demonstrated that family older adult care positively affected household income and wealth inequalities through mechanisms such as reducing labor participation of the household labor force and raising the threshold for entrepreneurship. However, compared with income inequality, the consumption inequality can better reflect the welfare levels of different households. Therefore, exploring the impact and transmission mechanism of care service supply on household consumption inequality can provide valuable insights.

The impact mechanism of family care service supply on income inequality is similar to that of household consumption inequality. As previously analyzed, family care service supply affects household consumption through the income and expenditure effect. Regarding the income effect, family care service supply reduces crowds out the number of working hours of household members, thus reducing income and generating household income inequality. Compared with the families with little or no care work, the difference of family care services causes income inequality among different families (34, 38). Regarding the expenditure effect, family care service supply affects household expenditure through household labor allocation, livelihood sources, and health level (39). Accordingly, this study argues that the potential impact mechanism of family care service supply on household consumption inequality is the “income effect of family care service supply.” Moreover, considering the particularity of “one old and one young” households, studies have rarely investigated the impact of care service supply on household consumption inequality considering minor child care by families. Accordingly, the third hypothesis is proposed as follows:

*H3*: Household income inequality is the mediating mechanism through which care service supply affects household consumption inequality; that is, there is an income inequality effect of household care service supply.

## 4 Methods and data

### 4.1 Approach

This paper uses Kakwani index to measure the consumption inequality index among households, and uses ordinary least squares (OLS) model for empirical test.

### 4.2 Design

#### 4.2.1 Computing consumption inequality

Many methods are available for measuring consumption inequality, such as the commonly used Gini coefficient and Theil index methods. However, these indicators have significant limitations when measuring individual or household income and consumption inequality, and cannot fully reflect the household-level income or consumption structure information. For example, Gini coefficient cannot reflect the specific structure of income distribution, nor can it distinguish the specific gap between different income groups. Therefore, when using Gini coefficient, we also need to combine other indicators for comprehensive analysis. Unlike Gini coefficient, Theil index can decompose the overall gap into inter group gap and intra group gap, so as to analyze the source of income inequality in a more detailed way. However, the Theil index is mainly used to measure the income gap between regions, which is not fully applicable to the measurement of other types of inequality.

This study is mainly to investigate the impact of care services on consumption inequality among different families, with the focus on different families. Therefore, we need to choose a more accurate micro household consumption inequality measurement method. Some typical micro-level measures of consumption inequality include the Kakwani or Zenga indices. Both indices exhibit excellent characteristics. The Zenga index is more sensitive to income or consumption inequalities of the low-income groups. Further, the Zenga inequality curve shows more details about the dynamic changes in differences. Meanwhile, the biggest characteristic of the Kakwani index is that after summation, it is closely related to the Gini coefficient. The Kakwani index reflects the relative deprivation index of individuals. Based on the characteristics and advantages of each index, this study uses the Kakwani index to measure household consumption inequality. According to Kakwani et al. (40), each household is compared with other households with higher household consumption expenditures, thus yielding inequality in household consumption expenditures. The index is computed as follows:


(1) $$RD\;\left(y,y_{i}\right)=\frac{1}{n{\bar{\mu }}_{Y}}\left(\overset{n}{\underset{j=i+1}{\sum }}\left(y_{j}-y_{i}\right)\right)=\gamma_{y_{i}}^{+}\left[\left(\mu_{y_{i}}^{+}-y_{i}\right)/{\bar{\mu }}_{y}\right]$$

Here, the reference group *Y* with n samples is ranked according to their consumption levels within the group, and then the overall consumption distribution function of the reference group, $Y=\left(y_{1},y_{2},.\ldots ,y_{n}\right)$, is obtained. Finally, Equation 1 is computed, which is the relative deprivation index of household consumption, or the consumption inequality index. $\mu_{Y}$ represents the average consumption of all household groups in group Y, $\mu_{yi}$ represents the average consumption of households that consume more than $y_{i}$ in group Y, and $\gamma_{yi}$ represents the percentage of samples that consume more than $y_{i}$ in group Y out of the total sample size.

#### 4.2.2 Design of empirical model

The following ordinary least squares estimation is used for regression analysis:


(2) $$\begin{array}{l} \mathrm{Consumption}\_\mathrm{inequalit}y_{i}=\alpha_{0}+\alpha_{1}\;\mathrm{Caregiving}+\\ \alpha_{2}CV_{i}+\mu_{i}+\varepsilon_{0} \end{array}$$

Here, in Equation 2, *Consumption_inequality_i_* represents household consumption inequality. This study also examines the differences in the impact of household care service supply on household consumption category inequality, or the inequality in the household consumption structure. According to the classification of household consumption categories, household food, alcohol, clothing, housing, etc., are defined as survival consumption; education, transportation and communication, medical care, etc., are defined as development consumption; and cultural and entertainment services, daily necessities and services, and other supplies and services are defined as enjoyment consumption. At the same time, although the statistics of household consumption in this paper are divided into three categories, the overall content does not include household care consumption. Its main body is divided according to the eight consumption categories published by the National Bureau of statistics of China, that is, it includes survival consumption, development consumption and enjoyment consumption. Based on the Kakwani index, their respective differences are estimated.

Caregiving represents the household care service supply. Care service supply includes two parts: family childcare and older adult care. These two parts are separately tested in the model, with the sign and size of coefficient $\alpha_{1}$ being of central interest.

CV_i_ represents individual characteristic variables. Following existing research (19, 20), this study controls individual and family characteristics. Individual characteristics include age, gender, marriage, self-rated health, household registration, presence of medical insurance, and formal labor force participation. Family characteristics include social status, economic status, and family size.

$\mu_{i}$ represents the regional fixed effect. Since this article uses cross-sectional data analysis, the time fixed effect has little impact. It is only the year difference in survey sample selection and has no practical significance. $\varepsilon_{0}$is a random error term. Robust standard errors are used in the regression analysis.

Next, this study uses a stepwise analysis method to test the mediating effect. The regression model is as follows.


(3) $$M_{i}=b_{0}+b_{1}\mathrm{Caregiving}+b_{2}CV_{i}+\mu_{i1}+\varepsilon_{1}$$

(4) $$\begin{array}{l} \mathrm{Consumption}\_\mathrm{inequalit}y_{i}=c_{o}+c_{1}\;\mathrm{Caregiving}+c_{2}M+\\ c_{3}CV_{i}+\mu_{i2}+\varepsilon_{2} \end{array}$$

Here, in Equations 3, 4 M is the mediating variable, which is the household income inequality. The focus is on coefficients b_1_, C_1_, and C_2_. When these are all significant, it indicates the existence of a mediating effect. When C_1_ is significant and b_1_ and C_2_ are not significant, it is necessary to test the significance of the product of the coefficients; that is, whether b_1_C_2_ = 0 should be rejected. A rejection of this null hypothesis indicates the existence of a mediating effect. When C_1_ is not significant but b_1_ and C_2_ are significant, it indicates the existence of a complete mediating effect; otherwise, it indicates a partial mediating effect.

### 4.3 Data

The data were selected from the 2018 China Family Panel Studies (CFPS) survey. The CFPS is implemented by the China Social Science Survey Center of Peking University. Its comprehensive survey questionnaire can comprehensively reflect the changes in China’s society, economy, population, education, and health. The sample survey collects community, family, and individual survey data. As a national and large-scale social survey data covering 25 Chinese provinces and approximately 16,000 households in each survey sample, the CFPS not only includes surveys on household economic and non-economic welfare, but also surveys on family relationships, population migration, and health. This study selected the 2018 survey data. Based on the core variables of the benchmark model, the data were matched using STATA15 by household and individual characteristics. The household ID was used as a recognition benchmark to match the corresponding individual ID. Some missing values were processed using deletion and mean substitution methods. Individual variables that could not be matched to household IDs were deleted; variables with missing values (*N* = 328; 3.09% of total sample) are replaced with mean values based on the data distribution of core variables. Some missing values (*N* = 69; 0.64% of the total sample) are removed, such as gender variables. In addition, no abnormal values, such as negative values, were found for the core explanatory variable of household consumption before processing. Before matching, the basic household consumption data ranged from 0 to 1.00e+08 yuan/year. Although zero indicates that household consumption expenditure is zero, this may mean that such households mainly rely on non-cash consumption, such as government material assistance for poor households. After matching, the range of household consumption was 400 to 2,019,360 yuan/year. This processing and matching yielded a sample valid sample of 10,632 households.

Descriptive statistics for the core variables are listed in Table 1. The average value of household consumption inequality reached 0.3530, indicating a relatively high degree of overall consumption inequality, with a maximum value of 0.9976. The average values for survival, development, and enjoyment consumption, and household income inequality were 0.3546, 0.4130, 0.5665, and 0.3959, respectively. The proportion of households with family care was 24.88%, of which only 3.42 and 21.45% were families taking care of children and older adults, respectively.

**Table 1:** Descriptive statistics.

Variable	Definition	Sample	Mean	S.D.	Min	Max
Household consumption inequality	Inequality in total annual household consumption computed by the Kakwani index	10,632	0.3530	0.3549	−8.1481	0.9976
Survival consumption inequality	Including food, tobacco, clothing, and housing, which are summed and estimated using the Kakwani index	10,632	0.3546	0.3532	−7.2973	0.9994
Developmental consumption inequality	Including education, transportation and communication, and healthcare, and the sum is calculated using the Kakwani index	10,632	0.4130	0.3651	−9.0496	0.9980
Enjoyment consumption inequality	Including cultural and entertainment services, household goods and services, and other goods and services, which are calculated after summing and using the Kakwani index	10,632	0.5665	0.3813	−9.5166	0.9997
Income inequality	Inequality index of total household income calculated using the Kakwani index	10,632	0.3959	0.3968	−8.0193	0.9999
Family care	Care for older adults over 60 years old or children under 18 years old = 1, no = 0	10,632	0.2488	0.4323	0	1
Childcare	Number of minors under 18 years old in the care of the family, have = 1, no = 0; unit: person	10,632	0.0342	0.1818	0	1
Older adult care	Care for 1 or more older adults over 60 years old = 1, no care = 0	10,632	0.2145	0.4105	0	1
Childcare frequency	Options 1–6 are almost one day a month, one day a month, 2–3 days a month, 1–2 days a week, 3–4 days a week, and every day, respectively. The value is calculated using the number of children in the family and frequency of care. The larger the value, the higher the frequency of care.	10,632	1.7837	4.1785	0	25
Older adult care frequency	Options 1–6 are almost one day per month, one day per month, 2–3 days per month, 1–2 days per week, 3–4 days per week, and every day, respectively. The sum of the number of older adults in the family and frequency of care is obtained. The larger the value from 0 to 12, the higher the frequency of care.	10,632	11.1141	2.4026	0	12
Family size	Total population of the family; unit: person	10,632	4.4221	2.0860	1	17
Medical insurance	Participation in basic medical insurance = 1, no = 0	10,632	0.9090	0.2877	0	1
Age	Actual interviewee age at the time of the survey, in years	10,632	36.8377	12.4703	18	96
Social position	Self-assessed social status rated 1–5, with higher values indicating higher status	10,632	2.9784	1.0096	1	5
Economic status	Self-assessed economic status rated 1–5, with higher values indicating higher status	10,632	2.8885	0.9949	1	5
Marital status	Unmarried, divorced, widowed, etc. = 0, married = 1	10,632	0.7903	0.4071	0	1
Household registration	Agricultural household registration = 0, non-agricultural household registration = 1	10,632	0.2595	0.4384	0	1
Gender	Female = 0, Male = 1	10,632	0.5088	0.4999	0	1
Per-capita income	Annual per capita income of the family, based on 2010 as the baseline, in units of ten thousand yuan	10,632	23,476	29,372	1.25	846,667
Education level	0 = never attended school; 1 = illiterate or semi-illiterate; 3 = primary school; 4 = junior high school; 5 = senior high/secondary technical/technical/vocational high school; 6 = college; 7 = bachelor’s degree; 8 = master’s degree; 9 = doctoral degree	10,632	4.1692	1.7244	0	9
Self-rated health	Self-rated health on 1–5 scale; higher value denotes better health	10,632	2.7421	1.0916	1	5
If participate in work	Participation in formal work, yes = 1, no = 0	10,632	0.7907	0.4068	0	1

## 5 Results

### 5.1 Benchmarking results

The regression results are presented in Tables 2, 3. Model (1) in Table 2 shows that caring for children has a significant positive effect on total household consumption inequality of 0.0023; that is, when the probability of caring for children increases by 1%, household consumption inequality increases by 0.23%. This may be because when the probability of family care for children increases, the family’s overall consumption level will decline, thus causing a consumption gap with families that have less pressures to care for children.

**Table 2:** Benchmark test results of inequality between childcare and household consumption.

Variable	Dependent variable: household consumption inequality			
	Total consumption (1)	Survival consumption inequality (2)	Developmental consumption inequality (3)	Enjoyment consumption inequality (4)
Childcare	0.0023*** (0.0006)	0.0029*** (0.0007)	0.0030*** (0.0007)	0.0035*** (0.0008)
Medical insurance (Yes = 1)	0.0098*** (0.0033)	0.0122*** (0.0036)	0.0081** (0.0039)	0.0123*** (0.0047)
Age	0.0006*** (0.0001)	0.0003*** (0.0001)	0.0007*** (0.0001)	0.0012*** (0.0001)
Social position	0.0045*** (0.0009)	0.0055*** (0.0010)	0.0039*** (0.0011)	0.0068*** (0.0014)
Economic status	−0.0007 (0.0009)	−0.0006 (0.0010)	−0.0009 (0.0011)	−0.0004 (0.0014)
Marital status (Yes = 1)	−0.0128*** (0.0023)	−0.0111*** (0.0026)	−0.0159*** (0.0027)	−0.0184*** (0.0032)
Household registration	−0.0075*** (0.0024)	−0.0214*** (0.0027)	−0.0054* (0.0028)	−0.0076** (0.0035)
Gender (Male = 1)	−0.0030* (0.0017)	−0.0013 (0.0019)	−0.0022 (0.0020)	−0.0056** (0.0025)
Family size	0.0092*** (0.0005)	0.0115*** (0.0005)	0.0100*** (0.0005)	0.0115*** (0.0007)
Per-capita income (2010 baseline)	0.0033*** (0.0006)	0.0053*** (0.0007)	0.0045*** (0.0007)	0.0045*** (0.0009)
Education level	0.0057*** (0.0006)	0.0091*** (0.0007)	0.0074*** (0.0007)	0.0064*** (0.0009)
Self-rated health	0.0010 (0.0007)	0.0017** (0.0008)	0.0006 (0.0009)	0.0011 (0.0010)
If participate in work	0.0033* (0.0020)	0.0008 (0.0023)	0.0002 (0.0024)	0.0037 (0.0028)
City fixed effect	Yes	Yes	Yes	Yes
Constant term	0.3669*** (0.0148)	0.3701*** (0.0158)	0.4097*** (0.0170)	0.5127*** (0.0189)
Observations	10,632	10,632	10,632	10,632
*R* ^2^	0.1692	0.2824	0.1845	0.1414

Robust standard errors are in parentheses; ***p < 0.01, **p < 0.05, *p < 0.1.

**Table 3:** Benchmark test results of inequality between older adult care and household consumption.

Variable	Dependent variable: household consumption inequality			
	Total consumption (1)	Survival consumption inequality (2)	Developmental consumption inequality (3)	Enjoyment consumption inequality (4)
Older adult care	0.0035*** (0.0012)	0.0031** (0.0013)	0.0050*** (0.0014)	0.0058*** (0.0017)
Medical insurance (Yes = 1)	0.0098*** (0.0033)	0.0123*** (0.0036)	0.0082** (0.0039)	0.0123*** (0.0047)
Age	0.0005*** (0.0001)	0.0002** (0.0001)	0.0006*** (0.0001)	0.0011*** (0.0001)
Social position	0.0044*** (0.0009)	0.0054*** (0.0010)	0.0037*** (0.0011)	0.0066*** (0.0014)
Economic status	−0.0008 (0.0009)	−0.0007 (0.0010)	−0.0009 (0.0011)	−0.0004 (0.0014)
Marital status (Yes = 1)	−0.0117*** (0.0023)	−0.0098*** (0.0026)	−0.0144*** (0.0027)	−0.0166*** (0.0032)
Household registration	−0.0074*** (0.0024)	−0.0213*** (0.0027)	−0.0053* (0.0028)	−0.0075** (0.0035)
Gender (Male = 1)	−0.0032* (0.0017)	−0.0015 (0.0019)	−0.0025 (0.0021)	−0.0059** (0.0025)
Family size	0.0090*** (0.0005)	0.0113*** (0.0005)	0.0097*** (0.0005)	0.0113*** (0.0007)
Per-capita income (2010 baseline)	−0.0034*** (0.0006)	−0.0054*** (0.0007)	−0.0046*** (0.0007)	−0.0045*** (0.0009)
Education level	−0.0058*** (0.0006)	−0.0091*** (0.0007)	−0.0074*** (0.0007)	−0.0065*** (0.0009)
Self-rated health	0.0010 (0.0007)	0.0018** (0.0008)	0.0007 (0.0009)	0.0013 (0.0010)
If participate in work	0.0034* (0.0020)	0.0009 (0.0023)	0.0003 (0.0024)	0.0039 (0.0028)
City fixed effect	Yes	Yes	Yes	Yes
Constant term	0.3843*** (0.0138)	0.3924*** (0.0144)	0.4316*** (0.0157)	0.5381*** (0.0174)
Observations	10,632	10,632	10,632	10,632
*R* ^2^	0.1693	0.2822	0.1848	0.1417

Robust standard errors are in parentheses; ***p < 0.01, **p < 0.05, *p < 0.1.

Regarding consumption categories, the results of models (2) to (4) in Table 2 indicate that caring for children has a significant positive effect on household survival, development, and enjoyment consumption inequalities, with corresponding effects of 0.29, 0.30, and 0.35%, respectively. Thus, as the probability of caring for children increases, it has stronger effects on high-level consumption inequality in households. Alternately, the crowding out effect of care labor for children in is mainly reflected in high-level consumption expenditure. Moreover, enjoyment consumption inequality is higher. Further, basic medical insurance, age, social status, marital status, household registration, family size, gender, household per-capita income, and educational level significantly affect household consumption inequality.

Table 3 reports the impact of older adult care on household consumption inequality. First, caregiving has a significant positive effect on total household consumption inequality, with an effect of 0.0035. That is, when the probability of family caregiving for older adults increases by 1%, total household consumption inequality will increase by 0.35%. Thus, older adult care weakens household consumption expenditure and increases consumption inequality. Second, regarding consumption categories, the results of models (2) to (4) in Table 3 show that caregiving for older adults significantly affects household survival, development, and enjoyment consumption inequalities by 0.31, 0.50, and 0.58%, respectively. Consistent with the results of childcare, the increase in the probability of caregiving for older adults also significantly damages high-level household consumption, with its impact on enjoyment-oriented consumption reaching a maximum of 0.58%. In addition, compared with the impact of childcare, the impact of older adult care is higher, indicating that the impact of family aging on household consumption will be stronger.

The results in Tables 2, 3 support hypothesis 1 that whereby the supply of care services, including care for older adults and children, has a significant negative effect on household consumption inequality. Essentially, consistent with previous research, the supply of care services increases household consumption inequality, resulting in differences in consumption levels between households with and without a care burden. Meanwhile, the results of models (2) to (4) in Tables 2, 3 support Hypothesis 2, which states that the supply of care services not only significantly affects household consumption inequality, but also significantly affects different types of household consumption inequality. Simultaneously, the impact on enjoyment-type consumption inequality is greater than that on survival-and development-type consumption inequalities.

In order to further clarify the source of the “crowding-out effect” of care services on household consumption inequality, on the basis of the benchmark test, we further investigated the conduction effect of family care service on different consumption categories, and the results are shown in Table 4. The consumption categories in Table 4 are divided into eight categories, that is, the eight categories of household consumption published by the National Bureau of statistics of China, in which the variables of each consumption category are taken as logarithms. In terms of childcare, the results show that childcare has a significant negative weakening effect on household expenditure variables of daily necessities and services, clothing variables, other daily necessities and services variables, while it has a significant positive effect on household EEC variables (Education, entertainment, culture services). Thus, when the family childcare service is increased by 1 unit, the family daily, dress and other variable will be significantly reduced by 2.65, 2.30 and 2.08%, respectively. The “crowding-out effect” is obvious, and the family EEC variable will be significantly increased by 6.95%. This result also shows that the childcare has a significant stimulating effect on the family education, entertainment, culture and other consumption expenditure. At the same time, caring for children has “crowding-out effect” on family food, house, med and trco variables, but none of them passed the significance test. In terms of care for older adults, Table 4 shows that care services for older adults have a significant positive effect on family EEC variables, med variables and trco variables, that is, when the family care services for older adults increase by 1 unit, family EEC consumption, med consumption and trco consumption will increase by 6.17, 7.64 and 3.89%, respectively. Care for older adults has a “crowding-out effect” on family daily, dress and house variables, but it has not passed the significance test. In general, the “crowding-out effect” of family consumption for caring for children is more obvious, while the “crowding-in effect” of family consumption for caring for the older adults is more obvious.

**Table 4:** Test results of crowding-out effect.

Variable	Dependent variable: household consumption							
	Daily (1)	Dress (2)	Eec (3)	Food (4)	House (5)	Med (6)	Trco (7)	Other (8)
Childcare	−0.0265*	−0.0230**	0.0695***	−0.0111	−0.0168	−0.0040	−0.0142	−0.0208*
	(0.0138)	(0.0098)	(0.0164)	(0.0080)	(0.0106)	(0.0124)	(0.0095)	(0.0124)
City fixed effect	Yes	Yes	Yes	Yes	Yes	Yes	Yes	Yes
Control variable	Control	Control	Control	Control	Control	Control	Control	Control
Observations	10,632	10,632	10,632	10,632	10,632	10,632	10,632	10,632
Adjust *R*^2^	0.1515	0.1930	0.1211	0.2678	0.0972	0.0818	0.2163	0.1756
Variable	Daily (1)	Dress (2)	Eec (3)	Food (4)	House (5)	Med (6)	Trco (7)	Other (8)
Older adult care	−0.0117	−0.0070	0.0617***	0.0007	−0.0217	0.0764***	0.0389***	0.0240
	(0.0179)	(0.0004)	(0.0190)	(0.0099)	(0.0145)	(0.0177)	(0.0109)	(0.0166)
City fixed effect	Yes	Yes	Yes	Yes	Yes	Yes	Yes	Yes
Control variable	Control	Control	Control	Control	Control	Control	Control	Control
Observations	10,632	10,632	10,632	10,632	10,632	10,632	10,632	10,632
Adjust *R*^2^	0.1512	0.1926	0.1202	0.2677	0.0971	0.0834	0.2169	0.1756

Robust standard errors are in parentheses. ***p < 0.01, **p < 0.05, *p < 0.1. The control variables are the same as those in Tables 2, 3. Daily = Household goods and services of daily life; Dress = Clothing etc; Eec = Education, entertainment, culture services, etc; Med = Medical care; Trco = Traffic communication; Other = Other goods and services.

### 5.2 Robustness test

To test the robustness of the benchmark results, this article replaces the explanatory variables, replaces the explained variables, restricts the sample, and uses the instrumental variable method.

#### 5.2.1 Replacing explanatory variable

The frequencies of childcare and older adult care are selected as alternative variables for household care service supply. The frequency of care reflects the specific intensity of the supply behavior of family care services, and this paper mainly examines it from the perspective of time intensity. The frequency of care includes two parts: the frequency of caring for children and the frequency of caring for the older adults. It is a further investigation of family care and reflects the impact of care duration on the overall consumption of the family. Therefore, the frequency of care service can more accurately reflect the impact of care services on family consumption inequality. The results in Table 5 using these alternative variables show that the main conclusions still hold. The higher the frequency of childcare (older adult care), the higher the household consumption inequality. Meanwhile, the impact of the frequency of older adult care on household consumption inequality is higher than that of childcare. Finally, by household consumption inequality categories, the frequency of care service supply has a greater impact on high-level consumption inequality as the household consumption level increases.

**Table 5:** Test results of replacing the core explanatory variables.

Variable	Dependent variable: household consumption inequality			
	Total consumption (1)	Survival consumption inequality (2)	Developmental consumption inequality (3)	Enjoyment consumption inequality (4)
Childcare frequency	0.0006***	0.0008***	0.0008***	0.0009***
	(0.0001)	(0.0002)	(0.0002)	(0.0002)
City fixed effect	Yes	Yes	Yes	Yes
Control variable	Control	Control	Control	Control
Observations	10,632	10,632	10,632	10,632
Adjust *R*^2^	0.1692	0.2825	0.1846	0.1415
Variable	Total consumption (5)	Survival consumption inequality (6)	Developmental consumption inequality (7)	Enjoyment consumption inequality (8)
Older adult care frequency	0.0009***	0.0010***	0.0016***	0.0017***
	(0.0003)	(0.0004)	(0.0004)	(0.0004)
City fixed effect	Yes	Yes	Yes	Yes
Control variable	Control	Control	Control	Control
Observations	10,632	10,632	10,632	10,632
Adjust *R*^2^	0.1715	0.2819	0.1839	0.1408

Robust standard errors are in parentheses. ***p < 0.01. The control variables are the same as those in Tables 2, 3.

#### 5.2.2 Replacing explained variables

Household per capita consumption is used instead of total household consumption to better capture average household consumption; accordingly, the corresponding household per capita consumption inequality index is computed and used as the explained variable. Note that as the data on household survival, development, and enjoyment consumption are based on households, they can also be converted into household per capita consumption after being divided by the number of household members. However, the consumption of each household member for each consumption category may differ and may not reflect the average household member’s consumption. Therefore, this study does not report the per-capita consumption inequality results for categories. The corresponding results after replacement are shown in Table 6. Both caring for children and caring for older adults have significant positive effects on household per capita consumption inequality of 0.19 and 0.26%, respectively. Thus, the benchmark test results still hold.

**Table 6:** Test results of replacing the core explanatory variable.

Variable	Dependent variable: inequality in household per capita consumption			
	(1)		(2)	
	Coefficient	Robust standard error	Coefficient	Robust standard error
Childcare	0.0019***	(0.0006)		
Older adult care			0.0026**	(0.0012)
City fixed effect	Yes		Yes	
Control variable	Control		Control	
Observations	10,632		10,632	
*R* ^2^	0.1755		0.2005	

Robust standard errors are in parentheses. ***p < 0.01,**p < 0.05. The control variables are the same as those in Tables 2, 3.

#### 5.2.3 Test of the dual care effect of “one old and one young” family

Due to the existence of “one old and one young” households, the benchmark results may not adequately reflect the dual pressures experienced by these households. Therefore, this article examines the impact of dual care on household consumption inequality by restricting the sample to these households. To achieve this, the older adult care and childcare variables are multiplied to generate a dual-care variable, where a final value of one indicates the existence of dual care; otherwise, it is a non-dual-care household. The results are listed in Table 7. Even under the pressure of dual care, the supply of care services still has a significant positive effect on total household consumption inequality, but its effect is only 0.05%. Meanwhile, its effects on household survival, development, and enjoyment consumption inequalities are only 0.04, 0.07, and 0.08%, respectively. Besides demonstrating the robustness of the main conclusions, this result also indicates that the impact of household care service supply on household consumption inequality is relatively low for “one old and one young” household. This may be because, on the one hand, many reasons may influence the impact of consumption inequality on such households, such as household income, family structure, and basic medical insurance, while care service supply only has a small one small impact. Indeed, the coefficients for these variables imply their stronger effects. On the other hand, care service supply has become inevitable work for all such households, which may mitigate the impact of care service supply on household consumption inequality.

**Table 7:** Test results of the impact of dual family care on household consumption inequality.

Variable	Dependent variable: household consumption inequality			
	Total consumption (1)	Survival consumption inequality (2)	Developmental consumption inequality (3)	Enjoyment consumption inequality (4)
Dual family care	0.0005***	0.0004**	0.0007***	0.0008***
	(0.0002)	(0.0002)	(0.0002)	(0.0002)
City fixed effect	Yes	Yes	Yes	Yes
Control variable	Control	Control	Control	Control
Observations	10,632	10,632	10,632	10,632
*R* ^2^	0.1693	0.2822	0.1848	0.1417

Robust standard errors are in parentheses. ***p < 0.01,**p < 0.05. The control variables are the same as those in Tables 2, 3.

#### 5.2.4 Addressing endogeneity concerns using the instrumental variable method

There may be endogeneity in the estimation results due to omitted variables, which may affect the robustness of the estimation results. To address this, this study uses the instrumental variable method. The duration of workday housework is selected as the instrumental variable for household care service supply. The duration of workday housework itself reflects the characteristics of family structure and personnel composition. For example, when a family has no children or older people to take care of, its housework may be relatively less, such as pure young families. When a family has only children or the older adults who need care, the overall duration of workday housework may increase, but when parents can help and do not need care, the duration of workday housework may decrease. Similarly, when a family needs the care of the older adults and children at the same time, when there is a serious shortage of staff, the caregivers’ duration of workday housework will inevitably increase. In addition, housework and family care are not exactly the same. The former focuses on daily household hygiene, cooking and other aspects, while the latter focuses on the daily life services of the family cared for. Therefore, on the whole, choosing the duration of workday housework can reflect the situation of family care to a certain extent. On the one hand, the duration of workday housework is not directly correlated with household consumption and household consumption inequality. On the other hand, the duration of workday housework can reflect the circumstances of household care service supply. For example, when a family must care for children or older adults, the duration of workday housework will be relatively higher, and vice versa. Therefore, the selected instrument meets the requirements for instrumental variables: the duration of workday housework is directly related to household care service supply, but not directly related to household consumption inequality. In the empirical regression, the duration of workday housework is log-transformed by adding 1. The results are listed in Table 8. The first-stage ordinary least squares (OLS) results of models (1) and (6) indicates that the duration of workday housework significantly affects the older adult and children care behaviors, demonstrating the effectiveness of the instrumental variable selection.

**Table 8:** Instrumental variable test results.

Variable	First-stage OLS	Second-stage 2SLS			
	Older adult care (1)	Total consumption (2)	Survival consumption inequality (3)	Developmental consumption inequality (4)	Enjoyment consumption inequality (5)
Childcare		0.0029* (0.002)	0.0036* (0.002)	0.0041** (0.002)	0.0086*** (0.002)
Instrumental variable	−0.7939*** (0.015)				
City fixed effect	Yes	Yes	Yes	Yes	Yes
Control variable	Control	Control	Control	Control	Control
Observations	10,632	10,632	10,632	10,632	10,632
*R* ^2^	0.0537	0.0511	0.1385	0.0228	0.0924

Variable	First-stage OLS	Second-stage 2SLS			
	Childcare (6)	Total consumption (7)	Survival consumption inequality (8)	Developmental consumption inequality (9)	Enjoyment consumption inequality (10)
Older adult care		0.0356*** (0.010)	0.0328*** (0.012)	0.0487*** (0.013)	0.0703*** (0.015)
Instrumental variable	0.1389*** (0.015)				
City fixed effect	Yes	Yes	Yes	Yes	Yes
Control variable	Control	Control	Control	Control	Control
Observations	10,632	10,632	10,632	10,632	10,632
*R* ^2^	0.3756	0.0990	0.1663	0.0935	0.0734

Robust standard errors are in parentheses. ***p < 0.01,*p < 0.1. The control variables are the same as those in Tables 2, 3. The explained variables in the table are total consumption and consumption inequality by category.

Next, the results of models (2) to (5) for the second-stage OLS regression in Table 8 indicate that caregiving for older adults still significantly affects household consumption inequality and consumption inequality categories; interestingly, its effect is higher than that in the benchmark results. The results of models (7) to (10) also indicate that caregiving for children still significantly affects household consumption inequality and its various categories; again, its effect is also higher than that in the benchmark results.

### 5.3 Group heterogeneity test

**Figure 2:**
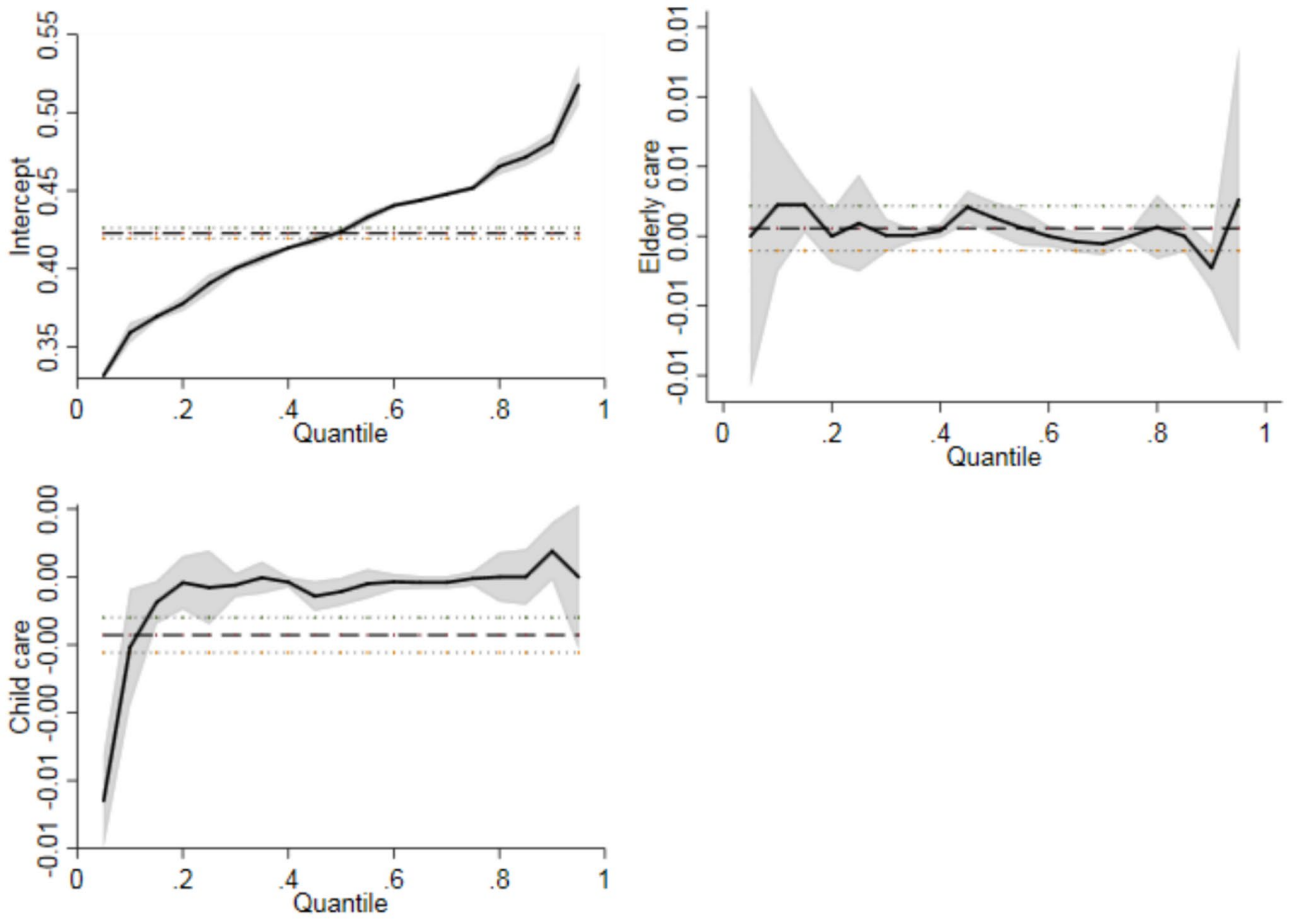
Group differences in the impact of care service supply on household consumption inequality.

Owing to differences in individual and family characteristics, the impact of care service provision on household consumption inequality may exhibit group heterogeneity. Therefore, this study further examines the differences in the impact coefficients by different household consumption inequality percentages. The results are shown in Figure 2. The overall coefficient distribution is relatively stable, but there are significant fluctuations at the lowest 10% and highest 10%. Moreover, the coefficient at the lowest 10% is relatively low, indicating that caring for older adults has little impact on households with the lowest consumption inequality. However, the coefficient at the highest 10% is relatively high, indicating that the impact of care service provision on households with the highest consumption inequality is higher. Next, in the quantile coefficient test results for caring for older adults, at the lowest 10% and highest 10% household consumption inequality percentages, the coefficient test has a wider shaded area in the 95% confidence interval. This implies that the test coefficients for caring for older adults in households with the lowest and highest household consumption inequality are unstable. This finding is consistent with the empirical results. Thus, compared to households with other consumption inequality levels, caring for older adults has obvious group differences in the impact on consumption inequality among households with the lowest and highest levels of consumption inequality.

In the quantile test results for childcare, Figure 2 shows significant group heterogeneity in the lowest 20% and highest 10% of household consumption inequalities. The impact coefficient of childcare on different household consumption inequalities shows a clear trend of first increasing and then stabilizing with fluctuations. When household consumption inequality is in the lowest 20% (highest 10%), the impact coefficient of childcare on household consumption inequality is the lowest (highest). However, the coefficient shows significant dispersion in the 95% confidence interval in the highest household inequality quantile, indicating that the estimation results for childcare in this quantile are less reliable. Therefore, there is significant group heterogeneity in the supply of care services in relation to household consumption inequality. The impact coefficient of care for older adults on household consumption inequality shows a relatively stable trend, whereas care for children shows a significantly rising trend. To effectively alleviate the impact of care service supply on household consumption inequality, it is necessary to pay attention to the supply of family care services at both extremes of consumption inequality and prioritize reducing the higher household consumption inequality caused by care service supply.

**Figure 3:**
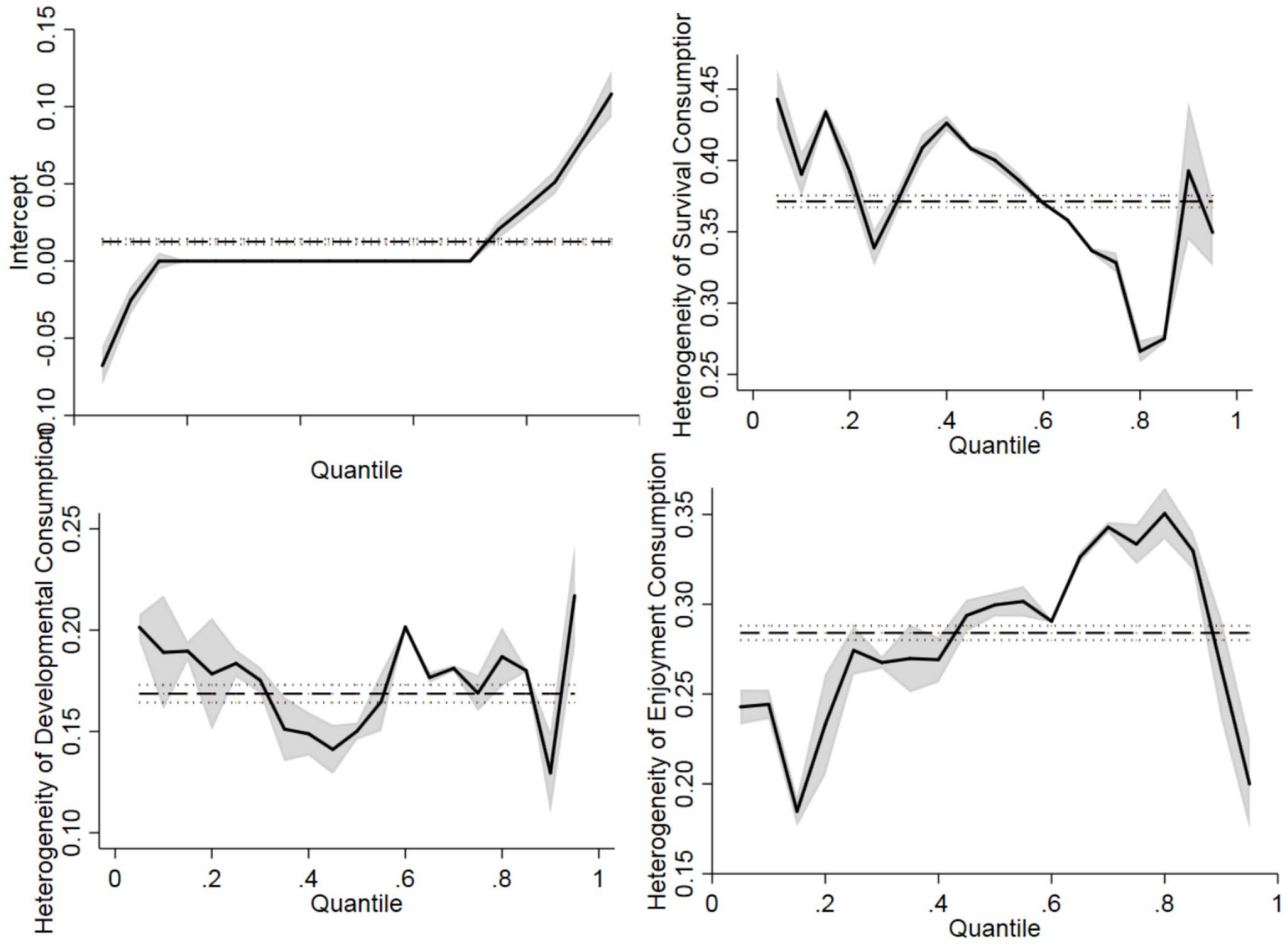
Differences in the impact of consumption inequality by category on total household consumption inequality.

Next, this study investigates the contribution coefficient of household consumption inequality categories to total household consumption inequality to test the rationality of the selection of classification consumption inequality indicators and their explanatory power for total consumption. The results are shown in Figure 3. The STATA coefficient test results generally provide the mean coefficients under different percentages, which makes it difficult to discern the differences in group influence coefficients under different percentages. Hence, this study chooses the quantile test. Figure 3 shows that, income inequality has a fluctuating trend on the impact of household consumption inequality under different percentages. Its impact coefficients on the lowest and highest percentages of household consumption inequality are the lowest and highest, respectively.

Overall, the impact of survival-oriented consumption inequality on total household consumption inequality under different percentages shows a fluctuating decreasing trend and exhibits high reliability under the 95% confidence interval, followed by an increasing trend above 80%. The impact of developmental consumption inequality on household consumption inequality under different percentages exhibits an inverted “W” shape;, compared to the two highest consumption inequality groups, its impact on the middle-income consumption inequality group is relatively lower, but nonetheless reaches a high level at 60%. The impact coefficient of enjoyment-oriented consumption inequality on total consumption inequality under different percentages shows a fluctuating increasing trend; however, after 80%, it exhibits a significant downward trend. Overall, compared to other households with different levels of consumption inequality, the impact of enjoyment-oriented consumption inequality on total household consumption in the 10–30 and 90% percentiles is relatively lower. In summary, the different types of household consumption inequality measurement indicators show differentiated trends in their impacts on total household consumption inequality under different percentages. Accordingly, policy interventions should be designed according to the different characteristics of influence to reduce the degree of consumption inequality among different groups or households.

### 5.4 Further analysis

Finally, this study explores the potential mechanism of the impact of care service supply on household consumption inequality. Care service supply directly affects household labor participation, which in turn impacts household income level, and thus, inequality. Therefore, one may argue that care service supply directly affects household income inequality, and which in turn affects household consumption inequality. This potential effect is termed as the “income inequality effect of family care service supply” and tested as the mediating mechanism. The results are summarized in Table 9. The results of models (1) to (5) indicate that childcare has a significant positive effect on household income inequality, whereas income inequality has a significant positive impact on total household, survival, development, and enjoyment consumption inequalities. Only in Model (3) does childcare have a significantly positive effect on survival consumption inequality. This shows that income inequality completely mediates the influence of childcare supply on total household, development, and enjoyment consumption inequalities; that is, there is a complete “income inequality effect of family care service supply.” In terms of survival consumption inequality, both the direct effect of caring for children and indirect mediating effect of income inequality exist. Thus, childcare influences household consumption inequality through the income inequality channel, thereby supporting Hypothesis 3.

**Table 9:** Mediating mechanism test results of the impact of care service on household consumption inequality.

Variable	First-stage OLS	Second-stage 2SLS			
	Household income (1)	Total consumption (2)	Survival consumption inequality (3)	Developmental consumption inequality (4)	Enjoyment consumption inequality (5)
Income inequality		0.6629***	0.6841***	0.7432***	0.9398***
		(0.0099)	(0.0101)	(0.0121)	(0.0139)
Childcare	0.0023***	0.0006	0.0011*	0.0010	0.0006
	(0.0006)	(0.0005)	(0.0006)	(0.0006)	(0.0007)
City fixed effect	Yes	Yes	Yes	Yes	Yes
Control variable	Control	Control	Control	Control	Control
Observations	10,632	10,632	10,632	10,632	10,632
*R* ^2^	0.2394	0.5839	0.6071	0.5643	0.5924

Variable	First-stage OLS	Second-stage 2SLS			
	Household income (6)	Total consumption (7)	Survival consumption inequality (8)	Developmental consumption inequality (9)	Enjoyment consumption inequality (10)
Income inequality		0.6631***	0.6845***	0.7432***	0.9398***
		(0.0099)	(0.0101)	(0.0121)	(0.0139)
Older adult care	0.0054***	−0.0001	−0.0006	0.0011	0.0005
	(0.0012)	(0.0008)	(0.0010)	(0.0010)	(0.0012)
City fixed effect	Yes	Yes	Yes	Yes	Yes
Control variable	Control	Control	Control	Control	Control
Observations	10,632	10,632	10,632	10,632	10,632
*R* ^2^	0.2401	0.5838	0.6071	0.5643	0.5924

Robust standard errors are in parentheses; ***p < 0.01, **p < 0.05. The control variables are the same as those in Tables 2, 3. The explained variables in the table are total consumption and consumption inequality by category.

The results of Models (6) to (10) in Table 9 indicate that caring for older adults significantly affects household income inequality, while household income inequality significantly affects total household consumption inequality and its different categories. This again supports the existence of mediating effects. However, older adult care in Models (2)–(5) has no significant impact on household consumption inequality, indicating that the mediating effect is complete. That is, household income inequality completely mediates the impact of older adult care on household consumption inequality and its different categories.

## 6 Discussion

This study examines the impact of care service supply for older adults and children on household consumption inequality and its categories, such as survival, development, and enjoyment consumption inequalities. The 2018 CFPS data are analyzed using the OLS regression, and several robustness tests are conducted, including replacing the explanatory and explained variables, restricting the sample, and using the instrumental variable method.

Research typically examines the economic effects of family caregiving for older adults or children from the perspectives of aging or childcare. However, with the development of the social economy and changes in family structure, the emergence of “one old and one young” families, where these families must care for both older adults and children, has significantly affected domestic or consumption demand in developing countries. Considering relatively low social welfare levels and heavy family burdens, an important concern for augmenting economic development in developing countries is how to better liberate family caregiving labor, and thus, enhance family consumption capacity. This article’s results show that family caregiving services for children and older adults significantly affect household consumption inequality, consistent with studies on household consumption (32, 41–44). Meanwhile, through the joint effect of dual care, this study demonstrates the differential effects of childcare and older adult care on household consumption. First of all, in terms of the differences of care service groups, the existing studies have paid too much attention to the impact of family consumption of care services for a certain type of older people (32, 43). Specifically, the consumption inequality effect of older adult care services exceeds that of childcare services. Furthermore, the impact of caregiving services on enjoyment consumption inequality is significantly higher than that on survival consumption inequality. Secondly, the supply of family care services is an important basis for the supply of social care services, and the latter is an important supplement to the former. However, the existing policies ignore the importance of the former, and the development of the latter is relatively lagging behind (45, 46). When social security or services are insufficient in a country or region, excessive dependence on family caregiving directly impacts household consumption inequality. Although caregiving labor itself also brings about direct survival consumption, it does negatively affect such households’ enjoyment consumption, thus hindering their overall quality of life. Meanwhile, the constraints of caregiving labor services on family labor hinder the social participation and income increase of the core labor force, thus affecting the development consumption of such households. This further reduces their survival consumption and enjoyment consumption levels, forming a new cycle of inequality. Eventually, a vicious cycle of “caregiving services-labor participation deficiency-development restriction-consumption downgrade” is formed.

Thirdly, families with multiple care burdens should be the priority focus of the government, but existing studies does not pay attention to the vulnerability of such families (44, 47, 48). This study also shows that compared families which only care for older adults or children, the burden of dual-caregiving families does not result in higher household consumption inequality. This may be because on the one hand, such families are already at a relatively high level of consumption. An increase in caregiving burden does not worsen their household consumption inequality. On the other hand, due to the heavy burden of caregiving in such households, and the transmission and scale effects of caregiving services, the care time investment in such households does not change significantly, thus showing a lower impact of consumption inequality.

Overall, regardless of whether a family is caring for older adults, children, or both, the socialization of care labor is becoming increasingly severe. Dependence on family care labor not only leads to insufficient formal work participation of the core family labor force, thereby reducing household income levels and consumption capacity, but also causes a low-level cycle of family care labor (46, 48, 49). Therefore, the types of family care burden must be examined as a whole (45, 47, 50, 51), starting from the categories of family care burden, to explore the mechanisms of the impact of care labor on household consumption inequality. These insights can help in better designing and promoting household income stability mechanisms and improvements in consumption capacities, and thus, enhance domestic demand, thereby contributing to the stable development of developing countries.

This study has the following advantages: First, using survey data from China, one the largest developing countries which is rapidly aging in a unique demographic context shadowed by the one child policy, this study explores the impact of care services on household consumption inequality and its transmission mechanism. This provides important empirical evidence for developing countries to better adjust social welfare policies and release household consumption power when faced with economic underdevelopment and an aging population. Second, in analyzing family care, this study not only examines single older adult or young families, but also focuses on the impact on dual care families, who must care for both older adults and children. Finally, this study also examines the differences in the impact of care services on different consumption inequality categories, including household survival, development, and enjoyment consumption inequalities. This provides a detailed picture of consumption inequality, thus enriching research on the economic effects of care labor.

This study has certain limitations. For example, this study only analyzed Chinese survey data; however, the applicability of the conclusions may be limited in developing countries with different development scales and cultural characteristics. In addition, due to the limitations of the survey samples, the situation of dual care families was examined not explicitly based sample of such families but based on the analysis of older adult and child care families. The formation paths of these three family types are relatively complex and were not considered in this study. For example, large families are likely to have a family structure with an older adult parent and a young child; they may have a better ability to smoothen the burden of care through internal economic strength. Therefore, there may be an estimation bias in the impact of care labor on consumption inequality in such families. Future research can explore related care and consumption data in other relevant developing countries. Scholars can also investigate the formation logics and paths of family structure to examine the underlying mechanisms.

This study’s findings have the following implications: First, considering that family care service supply for childcare and older adult care significantly affect household consumption inequality and different types of consumption inequality, high-quality social service must be urgently developed given the aging population and low fertility rate. On the one hand, social assistance policies for different family structures must be gradually improved as livelihood security policies. Furthermore, the social value of family care service supply should be considered important in policies, and financial compensation for family care service supply should be gradually increased to prevent families falling into difficulties due to care service supply. On the other hand, the complementarity of social and family care service supplies should be considered, such as accelerating the improvements in long-term care and socialized childcare services, to adapt to the needs of home care services in the today’s era.

Second, given the insufficient total demand for socioeconomic development in China, stimulating household consumption capacity is an important way to alleviate the country’s lack of economic development momentum. As the family care service supply limits household consumption and increases consumption inequality among different households, family care interventions based on social development policies are needed, the socialized development of older adult care and childcare service supply should be promoted, and the care services market should be developed. Guided by the market demand for care services, which is likely to remain strong into the future, this industry’s development can be improved. This can also help unleash demand from family caregivers and promote consumption vitality. Finally, targeted assistance policies need to be improved for households with dual care needs.

## 7 Conclusion

The findings and conclusions of this study are as follows: (1) Household care service supply, such as caring for children and older adults, significantly affects household consumption inequality. At the same time, the effect of the dual care pressure of “one old and one young” on household consumption inequality is relatively small, and it will face higher risks from the supply of care services.(2) The impact of caring for the older adults on household consumption inequality is significantly higher than that of caring for children. With the rise of consumption levels, the impact of family care service on consumption inequality at different levels is also increasing. (3) Under the pressure of “one old and one small” family dual care, the dual care behavior exacerbated the inequality of family consumption. (4) The supply of family care services has the effect of income inequality.

## Data Availability

The datasets presented in this study can be found in online repositories. The names of the repository/repositories and accession number(s) can be found below: https://opendata.pku.edu.cn/dataverse/CFPS.

## Footnotes

^1^Data from the 2018 report “Care Work and Care Jobs for the Future of Decent Work” by the International Labour Organization (ILO).

^2^2021 statistics data released by the National Bureau of Statistics. https://data.stats.gov.cn/easyquery.htm?cn=C01

^3^Data sourced from the Notice on Conducting Assessment of the Demand for Elderly Care and Standardizing Service Work (GWYF [2019] No. 48) issued by the National Health Commission on August 26, 2019.
